# Storage, Disposal, and Use of Opioids Among Cancer Patients in Central China: A Multi-Center Cross-Sectional Study

**DOI:** 10.1093/oncolo/oyae049

**Published:** 2024-04-01

**Authors:** Mi Zhang, Huili Chen, Chenxi Luo, Xuanxuan Wang, Liang Liu, Dongfang Wu, Hong Cheng

**Affiliations:** Department of Pharmacy, Zhongnan Hospital of Wuhan University, Wuhan, China; Department of Breast and Urological Radiation and Medical Oncology, Zhongnan Hospital of Wuhan University, Wuhan, China; School of Public Health, Wuhan University, Wuhan, China; Department of Pharmacy, Zhongnan Hospital of Wuhan University, Wuhan, China; Department of Pharmacy, Zhongnan Hospital of Wuhan University, Wuhan, China; Department of Pharmacy, Zhongnan Hospital of Wuhan University, Wuhan, China; Department of Pharmacy, Zhongnan Hospital of Wuhan University, Wuhan, China

**Keywords:** opioid medication, cancer pain, storage, disposal, use

## Abstract

**Objective:**

Unsafe opioid-related practices can lead to abuse, diversion, and accidental overdoses. In this study, we aimed to describe the patterns and beliefs regarding the storage, disposal, and use of opioids among Chinese patients with cancer in their home settings, which remain unclear.

**Methods:**

A multicenter cross-sectional survey was conducted in Hubei Province from October 2022 to June 2023. We collected information on the storage, disposal, and use of opioids among cancer pain inpatients in the oncology department. Logistic regression was used to estimate the factors associated with unsafe disposal and use of opioids.

**Results:**

The survey included 221 patients with a median age of 62 years. Only 3.2% stored their opioids under lock and key, and 49.8% were unaware of proper disposal methods. Nearly one-fifth (19.5%) reported having received information on the safe storage (14.0%) and/or disposal (10.0%) of opioids. A total of 44.3% reported unsafe use by sharing (1.8%), losing (4.1%), or taking opioids at a higher dose than prescribed (42.5%). Patients who did not receive information on the safe disposal of opioids (OR = 4.57, *P* = .0423), had a history of alcohol use (OR = 1.91, *P* = .0399), and used opioids other than morphine (OR = 2.31, *P* = .0461) had higher odds of unsafe disposal practices. Individuals with an associate degree/bachelor’s degree or above were less likely to dispose of (OR = 0.36, *P* = .0261) and use (OR = 0.31, *P* = .0127) opioids unsafely.

**Conclusion:**

A significant proportion of Chinese patients with cancer exhibit unsafe practices in the storage, disposal, and use of opioids. The study highlights an urgent need for implementing routine education programs and drug “take-back” initiatives to improve opioid-related practices.

## Teaser

Unsafe opioid-related practices can lead to abuse, diversion, and accidental overdoses. This article describes patterns and beliefs regarding the storage, disposal, and use of opioids among Chinese patients with cancer.

**Implications for Practice**

This is the first study to describe patterns and beliefs regarding opioid storage, disposal, and use among Chinese cancer patients. In this study, most patients were not adequately informed about appropriate storage or disposal practices, and a significant proportion exhibited unsafe patterns and beliefs regarding the storage, use, and disposal of opioids. This poses the potential risk of increased availability for non-medical purposes. Our findings emphasize the urgent necessity to develop guidelines for safe opioid-related practices and implement routine educational programs, as well as drug “take-back” initiatives, to enhance opioid-related practices in China.

## Introduction

Pain is a prevalent and excruciating symptom experienced by patients with cancer that significantly affects their overall quality of life. It is widely acknowledged that opioids play a crucial role in cancer pain management,^1^ and many cancer patients require long-term home use of opioid medications for pain relief.

All opioids inherently carry varying degrees of risk of abuse and are strictly regulated by countries worldwide. According to the latest published “Annual Report on Drug Abuse Monitoring (2016)” by the China Food and Drug Administration, the abuse of medical drugs accounted for 4% of the total drug abuse cases. Among these abused medical drugs, 53.4% were narcotic drugs, 42.9% were Class II psychotropic drugs, and 7.3% were class I psychotropic drugs, other prescription drugs and over-the-counter drugs. The most frequently abused medical drugs were methadone, morphine, diazepam, tramadol, and compound diphenoxylate. The main source of drug abuse among new drug abusers was “peer” (65%), followed by black market (15%) and entertainment locales (11.7%).^2^ Additionally, it has been reported that most individuals who abuse opioid medications obtain them from friends or relatives.^3^

To curb the illegal use of opioids in China, the government has implemented strict regulatory measures for the experimental research, manufacturing, commercial activities, transportation, and clinical administration of opioids. However, laws and administrative regulations pertaining to the control of narcotics and psychotropic drugs offer only limited guidance regarding the storage, disposal, and use of opioids in the home environment,^4-6^ despite the home being the primary care location throughout the course of the disease. Moreover, healthcare professionals rarely provide risk education on home storage, disposal, and use of opioids for patients with cancer pain. Due to disease progression and the development of tolerance to opioids, patients with cancer may require frequent rotation and dosage titration of opioids.^7^ Thus, there may be multiple unused or expired opioids in their homes, which exposes patients as well as their family members and friends to the risk of unsafe storage, improper disposal, and inappropriate use of opioids.

To the best of our knowledge, there is a dearth of data on opioid storage, disposal, use patterns, and beliefs among Chinese patients with cancer pain. Therefore, this survey study aimed to evaluate patterns and beliefs regarding the storage, disposal, and use of prescription opioids among patients with cancer pain in Hubei Province, central China. Additionally, this study aimed to identify the predictors of unsafe disposal and use of opioids.

## Method

This multicenter cross-sectional study was reviewed and approved by the Zhongnan Hospital of Wuhan University Institutional Review Board. Seventeen comprehensive hospitals in Hubei Province, central China, participated in this survey. We conducted random sampling of hospitalized patients admitted to the oncology department between October 1, 2022, and June 30, 2023. The inclusion criteria were as follows: (1) patients with cancer pain who were at least 18 years of age, (2) without cognitive impairments and proficient in spoken and written Chinese, (3) used prescription opioids for at least one month within the previous 12 months, and (4) the opioids were used at home. Patients who were unwilling to participate were excluded.

The questionnaire design employed adaptive logic, which means that the number of questions answered by respondents varied depending on their answers to specific questions. The survey consisted of a maximum of 38 questions, covering 4 aspects: demographic and clinical information, as well as the storage, disposal, and use of opioids. The questions were formulated based primarily on the recommendations by the Food and Drug Administration (FDA) and Drug Enforcement Agency (DEA) of the US, as well as Regulations on the Administration of Narcotic Drugs and Psychotropic Drugs (RANPD) of China. Additionally, previous studies on the safe storage, disposal, and use of prescription opioids were considered when formulating these questions. Upon completion of the questionnaire, patients were provided with educational material on the safe storage, disposal, and use of opioids.

To ensure completeness of the answers and enhance patient comprehension, we collected patient questionnaire responses through face-to-face interviews. At each sub-center, a clinical pharmacist was responsible for conducting the survey. During the interviews, the clinical pharmacist asked questions and filled out a questionnaire based on the patient’s responses. We standardized the interview process and developed a manual for questionnaire administration to ensure the homogeneity of the survey among the sub-centers. All investigators received standardized training and were aware of the research protocol before initiating the survey. Questionnaire interviews were conducted after obtaining written informed consent from the patients.

Our primary objective was to describe the patterns and beliefs regarding the storage, disposal, and use of opioids in patients with cancer pain in their home settings. Unsafe storage was defined as the storage of opioids in a manner other than under lock and key. Unsafe disposal was defined as the disposal of opioids by a method other than the officially recommended methods by either the FDA, DEA or RANPD. Unsafe use was defined as sharing, losing or taking opioids at a higher dose than prescribed. Our secondary objective was to identify predictors of unsafe opioid disposal and use.

The sample size was calculated using the following single proportion sample size estimation formula:


$$\begin{array}{l} N=\frac{Z_{1-\alpha /2}^{2}p\left(1-p\right)}{d^{2}}=\frac{1.96^{2}\times 0.91\times 0.09}{0.05^{2}}=125.85\approx 126 \end{array}$$

where *N* is the sample size, *Z* is the confidence interval (1.96), *p* is the proportion of safe storage of opioids (*p* = 0.09) obtained from a study conducted in the US,^8^ and *d* is the margin of error to be tolerated (0.05). By incorporating an additional 5% (126 × 0.05 = 6.3) of the sample size to compensate for non-respondents, the total required sample size was 133.

Statistical analysis was performed using R-version 4.3.1. The primary clinical outcomes were proportions of unsafe storage, disposal, and use of opioids. Descriptive statistics were used to summarize and compare the participant data. We used a logistic regression model to explore the associations between the primary outcomes and demographic information/patterns of opioid-related behavior. Univariate logistic regression analysis was conducted to identify the potential predictor of variables for the primary outcome, and then only the variables with a significance level of *p ≤ *0.20 were included in the multivariate logistic regression analysis.

## Results

**Figure 1.**
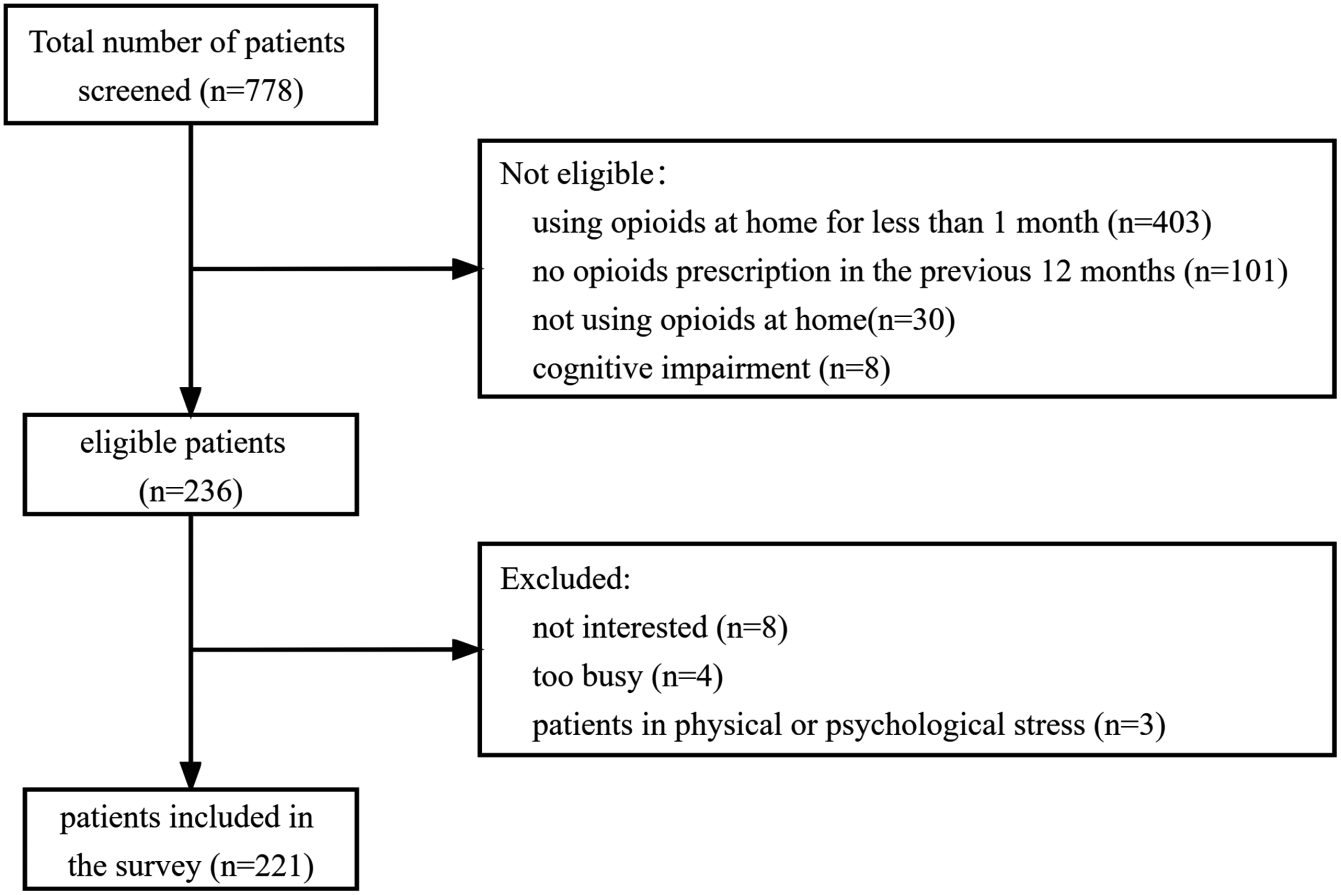
Flow chart of patient enrollment.

A total of 778 patients were screened, and 542 were ineligible due to factors such as the absence of an opioid prescription in the previous 12 months, not using opioids at home, using opioids at home for less than one month, or cognitive impairment. Of the 236 eligible patients, 15 declined to participate because they were not interested, too busy, or experienced physical or psychological stress. Ultimately, a total of 221 patients completed the survey. The flow chart of patient enrollment is shown in Figure 1. All participants completed ≥ 70% of the questions and were thus evaluable.

The demographic and clinical information of the patients is presented in Table 1. The median age was 62 years, 64.3% were male, 91.9% were married, and 95.9% lived with at least one other individual. Most respondents (62.4%) had a junior high school education or less, 23.5% had a high school education, 13.6% had an associate degree/bachelor’s degree or above, and only one patient (0.5%) had an advanced degree. Lung cancer (41.2%) was the most common primary cancer diagnosis, followed by hepatobiliary and pancreatic cancer (15.4%), and gastrointestinal cancer (14.9%). Prescriptions for products containing oxycodone (59.3%) or morphine (29.4%) were the most frequently reported opioids for cancer pain management, with a median oral morphine equivalent of 90.0 mg/day. CAGE (cut down, annoyed, guilty, and eye-opener questionnaire) screening was positive in 25.8% of respondents, and none of respondents reported a history of illicit drug use.

**Table 1.** Demographic and clinical information of patients.

Variable (*N* = 221)	Result
Age, median (IQR)	62 (55, 69)
Male, *n* (%)	142 (64.3)
Marital status, *n* (%)	
Married	203 (91.9)
Single/never married	3 (1.4)
Divorced	4 (1.8)
Widowed	8 (3.6)
Education, *n* (%)	
Junior high school and below	138 (62.4)
High school	52 (23.5)
Associate degree/bachelor’s degree	30 (13.6)
Advanced degree	1 (0.5)
Who lives at home, *n* (%)	
Parents	10 (4.5)
Spouse/significant other	169 (76.5)
Adult children/grandchildren (≥18 years)	72 (32.6)
Minor children/grandchildren (≤18 years)	13 (5.9)
Siblings	2 (0.9)
Alone	9 (4.1)
Other	1 (0.5)
Cancer type, *n* (%)	
Head and neck	11 (5.0)
Breast	10 (4.5)
Lung	91 (41.2)
Esophageal	5 (2.3)
Gastrointestinal	33 (14.9)
Hepatobiliary and pancreatic	34 (15.4)
Genitourinary	11 (5.0)
Gynecological	14 (6.3)
Sarcoma	1 (0.5)
Leukemia/lymphoma	3 (1.4)
Other	5 (2.3)
Opioid type, *n* (%)	
Morphine	65 (29.4)
Oxycodone	131 (59.3)
Fentanyl transdermal patches	36 (16.3)
Codeine	23 (10.4)
Tramadol	35 (15.8)
History of alcohol, *n* (%)	75 (33.9)
CAGE positive, *n* (%)	57 (25.8)
History of illicit drug use, *n* (%)	0 (0.0)
History of smoking, *n* (%)	82 (37.1)
MEDD (mg), median (IQR)	90.0 (40.1, 175.0)
Good medication adherence, *n* (%)	52 (23.5)

Abbreviations: IQR, interquartile range; CAGE, cut down, annoyed, guilty, eye-opener questionnaire; MEDD, morphine equivalent daily dose; medication adherence: evaluated by Medication Adherence Questionnaire (MAQ).^9^

Table 2 presents information on patterns and beliefs regarding opioid storage, disposal, and use. The vast majority of respondents (95.5%) had an unsafe opioid storage practice of storing opioids in plain sight or concealed locations without locks. Among those who did not store their opioids under lock and key, the primary reasons included not considering it necessary (62.6%) and having no concerns about medication theft (49.5%).

**Table 2.** Patterns and beliefs of opioid storage, disposal, and use.

Variable (*N* = 221)	*n*	%
*Storage of opioid medication*		
Where do you most often keep your opioids at home?		
Where everyone can see	63	28.5
Hidden but not locked	148	67.0
Under lock and key	7	3.2
Other	3	1.4
What are the reasons for not locking opioids for storage? (*n* = 214)		
Do not think it is necessary	134	62.6
Not worried about someone using my medication	106	49.5
Inconvenient to use	83	38.8
Never considered/heard about locking opioids for storage	76	35.5
Other	1	0.5
Have you received education or counselling on safe storage of opioids?		
Received no information	190	86.0
Received information (source of information)	31	14.0
Medication instructions/package insert	5	16.1
Physician	23	74.2
Nurse	13	41.9
Pharmacist	7	22.6
Newspapers, television, the Internet and other media	2	6.5
Other	3	9.7
*Disposal of opioid medication*		
I have leftover opioids at home.	26	11.8
I routinely dispose of opioids that I no longer need.	57	25.8
I keep the leftover opioids in case I need them in the future (*n* = 164)	123	75.0
What do you believe is the correct method for disposing of opioids?		
Give it back to doctor or pharmacy for disposal	103	20.6
Flush down the toilet	26	11.8
Throw it into the trash	82	37.1
Throw out in the trash after mixing (with cat litter, dirt, etc.) to prevent further use	49	22.2
Sell/give to others in need	4	1.8
Do not know/never thought about it	26	11.8
I have heard of the opioid “take-back” program	28	12.7
Have you received education or counselling on proper disposal of opioids?		
Received no information	199	90.0
Received information (source of information)	22	10.0
Medication instructions/package insert	4	18.2
Physician	12	54.5
Nurse	9	40.9
Pharmacist	9	40.9
Newspapers, television, the Internet and other media	3	13.6
Other	1	4.5
*Use of opioid medication*		
I only take opioids when the pain is unbearable.	104	47.1
I have lost my opioids in the past.	9	4.1
I tell others I am taking opioids.	45	20.4
My friends or relatives have asked me for my opioids.	4	1.8
I have shared my opioids with others.	4	1.8
I am likely to allow my family members or friends to use my opioid medication in the future.	26	11.8
How often do you take more opioid medication than prescribed by the doctor?		
Never	127	57.5
Rarely	70	31.7
Sometimes	21	9.5
Frequently	2	0.9
Always	1	0.5
I am aware that one dose of my current opioids can be fatal if taken by someone else	71	32.1

Approximately one in 10 respondents (11.8%) reported having leftover opioids at home. Among those with leftover opioids, one-quarter (25.8%) routinely disposed of them. The primary reason for retaining unused opioids was to save for future use if pain occurs (75.0%), followed by not knowing how to dispose of them properly (27.4%). Notably, half of the respondents (49.8%) were unaware of proper disposal methods for opioids. One-third (37.1%) of the patients perceived throwing opioids into the trash as an appropriate disposal method, 20.6% believed returning them to their doctor or pharmacy was the correct approach, and 11.8% admitted to lacking knowledge regarding proper disposal methods or never having contemplated it. Only 12.7% of patients had heard of opioid “take-back” programs.

Nearly one-fifth (19.5%) of the participants reported having received information on the safe storage (14.0%) and/or disposal (10.0%) of their opioids, whereas 80.5% had received neither storage nor disposal information. With regard to opioid use, 44.3% reported unsafe practices such as having shared opioids (1.8%), lost opioids (4.1%), or taken opioids at a higher dose than prescribed (42.5%). Additionally, nearly 11.8% of the respondents were likely to allow family members or friends to use their opioids in the future, and 67.9% were unaware that one dose of opioids can be fatal if taken by someone else.

Variables with *p ≤ *0.20 in the univariate logistic regression analysis of unsafe disposal/use are presented in Tables 3 and 4, respectively. As shown in Table 3, the multivariate regression model revealed that patients who had not received education or counseling on the safe disposal of opioids (OR = 4.57, *p* = 0.0423), had a history of alcohol consumption (OR = 1.91, *p* = 0.0399), and used opioids other than morphine (OR = 2.31, *p* = 0.0461) had higher odds of unsafe disposal practices. Furthermore, individuals with an associate degree/bachelor’s degree or above or above were less likely to engage in unsafe disposal practices (OR = 0.36, *p* = 0.0261). Table 4 indicates a lower likelihood of unsafe opioid use patterns among patients with an associate degree/bachelor’s degree or above (OR = 0.31, *p* = 0.0127). Conversely, patients who expressed willingness to allow their family members or friends to use their opioid medications in the future displayed an increased likelihood of unsafe opioid use (OR = 2.54, *p* = 0.0434).

**Table 3.** Predictors of unsafe opioid disposal.

Variable	Unsafe disposal	Safe disposal	Unsafe disposal, univariate logistic regression			Unsafe disposal, multivariate logistic regression		
			OR	95% CI	*P-*value	OR	95% CI	*P-*value
Have heard of the opioid “take-back” programs, *n* (%)								
Yes	6 (21.4)	22 (78.6)	1.00			1.00		
No	102 (53.7)	88 (46.3)	4.25	1.75-11.97	0.0027	2.07	0.66-6.93	0.2148
Received education or counselling on safe disposal of opioids, *n* (%)								
Yes	3 (13.6)	19 (86.4)	1.00			1.00		
No	107 (54.0)	91 (46.0)	7.45	2.44-32.42	0.0016	4.57	1.15-23.52	0.0423
Education, *n* (%)								
Junior high school and below	79 (57.7)	58 (42.3)	1.00			1.00		
High school	21 (40.4)	31 (59.6)	0.50	0.26-0.95	0.0350	0.62	0.31-1.24	0.1788
Associate degree/bachelor’s degree or above	10 (32.3)	21 (67.7)	0.35	0.15-0.78	0.0126	0.36	0.14-0.87	0.0261
History of alcohol use, *n* (%)								
No	66 (45.5)	79 (54.5)	1.00			1.00		
Yes	44 (58.7)	31 (41.3)	1.70	0.97-3.00	0.0655	1.91	1.04-3.58	0.0399
Age, median (IQR)	62 (55, 68)	63 (55, 70)	1.02	0.99-1.04	0.1508	1.01	0.98-1.03	0.6184
Who lives at home, *n* (%)								
Alone	6 (60.0)	4 (40.0)	1.00			1.00		
With minor children/grandchildren (<18 years)	4 (30.8)	9 (69.2)	0.30	0.05-1.60	0.1678	0.59	0.07-4.31	0.6077
Other	100 (50.8)	97 (49.2)	0.69	0.17-2.48	0.5705	0.72	0.13-3.18	0.6710
Opioid type, *n* (%)								
Morphine	13 (39.4)	20 (60.6)	1.00			1.00		
Other	97 (51.9)	90 (48.1)	1.66	0.79-3.60	0.1892	2.31	1.03-5.41	0.0461

Unsafe disposal is defined as the disposal of opioids by a method other than officially (FDA, DEA or RANPD) recommended.

Abbreviations: CI, confidence interval; IQR, interquartile range; OR, odds ratio.

**Table 4.** Predictors of unsafe opioid use.

Variable	Unsafe use	Safe use	Unsafe use, univariate logistic regression			Unsafe use, multivariate logistic regression		
			OR	95% CI	*P*-value	OR	95% CI	*P*-value
Education, *n* (%)								
Junior high school and below	68 (49.3)	70 (50.7)	1.00			1.00		
High school	22 (42.3)	30 (57.7)	0.75	0.39-1.43	0.3918	0.73	0.37-1.41	0.3521
Associate degree/bachelor’s degree or above	7 (23.3)	23 (76.7)	0.31	0.12-0.74	0.0124	0.31	0.11-0.74	0.0127
I am likely to allow my family members or friends to use my opioid medication in the future, *n* (%)								
No	80 (41.5)	113 (58.5)	1.00			1.00		
Yes	16 (61.5)	10 (38.5)	2.26	0.99-5.40	0.0572	2.54	1.05-6.52	0.0434
MEDD (mg), median (IQR)	95.0 (40.0, 160.0)	95.0 (40.0, 160.0)	1.00	0.99-1.00	0.1192	1.00	0.99-1.00	0.0979
Marital status, *n* (%)								
Married	91 (45.0)	111 (55.0)	1.00			1.00		
Other	4 (26.7)	11 (73.3)	0.44	0.12-1.35	0.1760	0.37	0.10-1.15	0.1049

Unsafe use is defined as having shared, lost or taken opioids at higher doses than prescribed.

Abbreviations: CI, confidence interval; IQR, interquartile range; MEDD, morphine equivalent daily dose; OR, odds ratio.

## Discussion

To the best of our knowledge, this is the first study to describe the patterns and beliefs regarding opioid storage, disposal, and use among patients with Chinese cancer. Our survey suggests that the current practices related to the storage, disposal, and use of opioid medications among Chinese patients with cancer are suboptimal. Only 3.2% stored their opioids under lock and key, 49.8% were unaware of the proper disposal methods for opioids, and 44.3% had shared, lost, or taken opioids at higher doses than prescribed.

Consistent with overseas research,^8,10-13^ only a minimal number of patients (3.2%) safely stored their opioid medications, although a significant majority (95.9%) resided with at least one other individual. This finding could partially be explained by the fact that only 14.0% of the patients received information on the safe storage of opioid medications. Moreover, unlike in the US, where drug storage information is readily available to the general public,^14-17^ there is no official recommended method for the home storage of opioid medications in China. The absence of official guidance has resulted in healthcare professionals and patients paying little attention to the safe storage of opioid medications.

Regarding opioid disposal, according to relevant regulations, patients are advised to return empty ampoules and waste patches to the pharmacy when they need to be prescribed an injection or patch for narcotic drugs. Similarly, when patients no longer need narcotic drugs, it is recommended to give leftover drugs to the pharmacy for disposal.^6^ Unfortunately, we found that one-third (37.1%) of patients perceived throwing opioids into the trash as an appropriate disposal method, while 20.6% believed returning them to their doctor or pharmacy was the correct approach, and only 12.7% of patients had heard of opioid “take-back” programs. This can be attributed to the following reasons: First, our survey revealed that patients who had not received education or counseling on the safe disposal of opioids were significantly more likely to dispose of opioids improperly (OR = 4.57, *p* = 0.0423). However, in contrast to previous studies conducted abroad which reported that 54.7% of the respondents had received information regarding proper opioid disposal methods,^10^ our survey findings indicated that only 10% of the participants reported being informed about safe opioid disposal practices. Second, patients believed that simply throwing leftover medication into the trash was the most cost-effective and hassle-free way to dispose of them, while sending them to hospitals for disposal was considered costly. Given the potential risks of increased abuse and diversion of opioids owing to improper disposal, it is therefore essential to establish simple and accessible drug disposal protocols. This can include implementing “take-back” sites in social retail pharmacies, organizing periodical community “take-back” programs, and following FDA recommendations such as flushing drugs down the toilet or mixing them with dirt before disposing of them in the trash.^18-22^ Lastly, the medical information system in China is not networked for prescribing and dispensing narcotic drugs, thus, patients can obtain the same prescription from different hospitals. As a result, it is not mandatory to return their empty ampoules and waste patches for a new narcotic drug prescription. Ultimately, establishing information interconnection for narcotic drugs is essential to avoid repeated prescriptions in different hospitals and reduce the medication risk for patients.

Our survey findings suggest that patients who used opioid other than morphine (OR = 2.31, *p* = 0.0461) exhibited a higher likelihood of unsafe disposal practices. This observation can be attributed to the widespread use of morphine as an opioid analgesic, which has resulted in greater public awareness of the potential hazards associated with its misuse compared to other opioids in China.

Educational attainment also plays a role in influencing opioid-related practices, as evidenced by the finding that patients with an associate degree/bachelor’s degree or above exhibited a lower likelihood of unsafe disposal (OR = 0.36, *p* = 0.0261) or unsafe use (OR = 0.31, *p* = 0.0127). The median age of the respondents was 62 years, with the majority born between the 1950s and the 1960s. This cohort generally exhibited lower educational attainment,^23^ which aligns with our survey findings indicating that 85.9% of the participants had completed high school education or below. Consequently, the combination of limited education, inadequate scientific literacy, and insufficient information on opioids contributes to a lack of awareness of the associated risks, leading to unsafe disposal and usage patterns or beliefs. Furthermore, we were astonished to find that more than half of the patients (67.9%) were unaware that a single dose of opioid medication can be fatal if taken by someone who has not been prescribed opioids. This highlights the significant lack of awareness of the risks associated with opioid use, which may contribute to unsafe opioid-related practices.

Our results revealed that patients with cancer pain treated with opioids need to be educated about home storage, disposal, and usage of opioids to mitigate the risk of diversion and accidental poisoning. Patient education, conducted by physicians during prescriptions and by pharmacists or nurses during dispensing, has demonstrated efficacy in enhancing knowledge and inducing behavioral changes regarding appropriate storage, disposal, and utilization of opioids.^24-26^ However, it is crucial to acknowledge that educating patients about medication storage and disposal should not be confined solely to opioids. As highlighted by Buffington et al, patient’s adherence to the routine practice of disposing of all unused prescription medications was the most influential factor leading patients to dispose of unused opioids.^27^ This insight emphasizes the importance of providing patients with information pertaining to proper storage and disposal practices for all prescribed medications, thereby substantially enhancing the overall medication safety within households.

Our study had several limitations. First, this survey is administered by clinical pharmacists rather than filled in by the patients discretely, which may have been subject to social desirability biases and lead to underreporting of unsafe opioid related practices. Additionally, our multicenter study was conducted in one province, therefore, we cannot confirm whether the sample is representative of all patients with cancer pain nationwide who use opioids. Finally, causality could not be determined due to the inherent nature of cross-sectional studies. Further research is required to confirm these findings.

## Conclusion

Current practices related to the storage, disposal, and use of opioid medications among Chinese patients with cancer pain should be improved to decrease the potential for medication misuse, diversion, and unintentional exposure. It is crucial to develop comprehensive and feasible guidelines that facilitate the safe storage and proper disposal of opioids while ensuring easy accessibility for the general public. Additionally, it is recommended to incorporate routine educational programs that emphasize the significance of safe opioid-related practices during prescription administration, along with providing easily accessible methods for proper opioid medication disposal.

## Data Availability

The data underlying this article will be shared on reasonable request to the corresponding author.
